# Mucocutaneous Leishmaniasis/HIV Coinfection Presented as a Diffuse Desquamative Rash

**DOI:** 10.1155/2014/293761

**Published:** 2014-12-08

**Authors:** Guilherme Almeida Rosa da Silva, Daniel Sugui, Rafael Fernandes Nunes, Karime de Azevedo, Marcelo de Azevedo, Alexandre Marques, Carlos Martins, Fernando Raphael de Almeida Ferry

**Affiliations:** ^1^Federal University of the State of Rio de Janeiro (UNIRIO), 20550-110 Urca, RJ, Brazil; ^2^Federal University of Rio de Janeiro (UFRJ), 20550-110 Urca, RJ, Brazil; ^3^Porto University, 4099-002 Porto, Portugal

## Abstract

Leishmaniasis is an infectious disease that is endemic in tropical areas and in the Mediterranean. This condition spreads to 98 countries in four continents, surpassing 12 million infected individuals, with 350 million people at risk of infection. This disease is characterized by a wide spectrum of clinical syndromes, caused by protozoa of the genus *Leishmania*, with various animal reservoirs, such as rodents, dogs, wolves, foxes, and even humans. Transmission occurs through a vector, a sandfly of the genus *Lutzomyia*. There are three main clinical forms of leishmaniasis: visceral leishmaniasis, cutaneous leishmaniasis, and mucocutaneous leishmaniasis. The wide spectrum of nonvisceral forms includes: localized cutaneous leishmaniasis, a papular lesion that progresses to ulceration with granular base and a large framed board; diffuse cutaneous leishmaniasis; mucocutaneous leishmaniasis, which can cause disfiguring and mutilating injuries of the nasal cavity, pharynx, and larynx. Leishmaniasis/HIV coinfection is considered an emerging problem in several countries, including Brazil, where, despite the growing number of cases, a problem of late diagnosis occurs. Clinically, the cases of leishmaniasis associated with HIV infection may demonstrate unusual aspects, such as extensive and destructive lesions. This study aims to report a case of mucocutaneous leishmaniasis/HIV coinfection with atypical presentation of diffuse desquamative eruption and nasopharyngeal involvement.

## 1. Introduction

Iconographic records of nonvisceral leishmaniasis (NVL) are primarily known to belong in the ceramics of pre-Inca Peru and Ecuador (400–900 AD). In these objects, we can observe the mutilated facies with characteristic mucocutaneous leishmaniasis (MCL) noses. In the Old World (Asia, Africa, and Europe) the written reports of the disease are dated from the first century AD [1]. Only a thousand years later, in 1903, the agent of the disease was first described, separately by Leishman and Donovan. A protozoan was identified in spleen tissue of two patients residing in India affected by a fatal disease. The disease in question was visceral leishmaniasis (VL) and the agent identified was* Leishmania donovani* [2].

Leishmaniasis is included in the group of neglected tropical diseases and, according to the World Health Organization (WHO) and the Laboratory of Tropical Diseases (LTD), considered one of the six most important diseases in the world [2, 3]. It is endemic in the tropics and in the Mediterranean, being spread by 98 countries in four continents with a total of 12 million infected people and 350 million at risk of infection. It is estimated that approximately 0.2 to 0.4 million of new VL cases and 0.7 to 1.2 million of new NVL cases occur each year worldwide [1, 4, 5].

From 2005 to 2009, a total of 96,351 cases of NVL and 13,563 cases of VL were registered in Brazil [6]. Between 2001 and 2011, 38,808 VL cases were recorded in the Americas. Although these cases are distributed in 12 countries, including recent reports from Paraguay and Argentina, 96% of American VL cases occur in Brazil (37,503 cases). In 2001, approximately 0.7% of all VL cases were reported in HIV-infected patients, while in 2012 this percentage increased to 8.5%. Epidemiological studies on NVL/HIV coinfection in Latin America are needed [7].

This disease is characterized by a wide spectrum of clinical syndromes caused by obligate intracellular protozoa of the genus* Leishmania*, from Trypanosomatidae family.* Leishmania* has mammal reservoirs extending to rodents, dogs, wolves, foxes, and even humans [2, 4]. Transmission occurs through a vector, a sandfly of the genus* Lutzomyia*, with* Lutzomyia longipalpis* and* Lutzomyia intermedia *currently known. Leishmaniasis is endemic where the vector, the animals that act as hosts, and reservoirs of the disease are found [8].

There are three main clinical forms of leishmaniasis: cutaneous leishmaniasis (CL), mucocutaneous leishmaniasis (MCL), capable of producing disfiguring injuries and severe scarring, and visceral leishmaniasis (VL) or “kala-azar” that is fatal without treatment. The NVL is present in 82 countries, with an annual incidence of 1.5 million cases worldwide. Several clinical forms are possible depending on the species of* Leishmania* involved [9]. Thus, we have CL and their forms: localized cutaneous leishmaniasis (LCL), papular lesions that progress to ulceration with a granular bottom and an edge with a high frame, with spontaneous cure in most cases; diffuse cutaneous leishmaniasis (DCL), clinically similar to leprosy, with symptoms difficult to treat; and MCL, which is the most severe form, which can cause disfiguring and mutilating injury of the nasal cavity, pharynx, and larynx. Furthermore, post-kala-azar dermal leishmaniasis (PKDL) form is not named as a clinical form within the cutaneous group and should be also considered [10].

A single species of* Leishmania* may produce signs and symptoms of various syndromes, and similarly each syndrome can be caused by several different species [9]. The patient's symptoms are the result of parasitic factors (invasion tropism and pathogenicity) and host immune cell-mediated replies [11]. In Brazil, the NVL agents documented are* Leishmania (Viannia) braziliensis*,* Leishmania (Viannia) guyanensis*,* Leishmania (Viannia) lainsoni*,* Leishmania (Viannia) naiffi*,* Leishmania (Viannia) shawi*,* Leishmania (Viannia) lindenbergi*, and* Leishmania (Leishmania) amazonensis* [12].

As leishmaniasis/HIV coinfection, HIV infection increases the risk of developing leishmaniasis between 100 and 1000 times, reduces the likelihood of therapeutic response, and greatly increases the risk of relapse [15]. At the same time, leishmaniasis accelerates the clinical course of HIV infection leading to AIDS earlier. Atypical presentations of leishmaniasis may arise by leishmaniasis/HIV coinfection; for example, there are reports of involvement of the viscera with NVL and skin compromise with VL [13]. Intravenous drug users are the main risk group of leishmaniasis/HIV coinfection in European southwest, constituting over 70% of coinfected patients [11, 13]. In Brazil, drug addicts are only 7% of the coinfected [14]. Epidemiological data shows that drug addicts are a population at risk, with proved existence of* Leishmania* parasitemia in asymptomatic blood donors from endemic areas [1]. Parasites were also detected in peripheral blood and in the skin of 88% of immunocompromised patients coinfected with leishmaniasis/HIV [15]. These data support the hypothesis that parenteral transmission of leishmaniasis man to man can occur [1].

We report a case of a patient with leishmaniasis/HIV coinfection who presented with an extensive and aggressive mucocutaneous form whose diagnosis established was MCL.

## 2. Case Report

This was a 29-year-old female patient, born in São Benedito, Ceará, ex-peasant, resident of Complexo do Alemão, Rio de Janeiro, Brazil, for 9 years. Denied recent travel history, intravenous drug use, or previous history of leishmaniasis.

The patient arrived with history of high fever, weight loss, night sweats, cough with yellowish secretion, generalized lymphadenopathy, and pain in the left hypochondrium. The patient sought outpatient medical care at the health center, with diagnosis of pulmonary tuberculosis and treatment with rifampicin, isoniazid, pyrazinamide, and ethambutol.

Five months after treatment, she returned to the health center presenting with painless erythematous punctate lesions on the face without pruriency, associated with fever, vomiting, and watery diarrhea. Besides this, she complained of severe and disabling headache. She was referred to Gaffrée and Guinle Universitary Hospital for diagnostic evaluation. Cranial CT scan evidenced lesions consistent with toxoplasmosis. HIV screening test was positive. She was hospitalized with oral prescription of sulfadiazine 4 g/day, pyrimethamine 75 mg/day, and folinic acid 15 mg/day.

**Figure 1:**
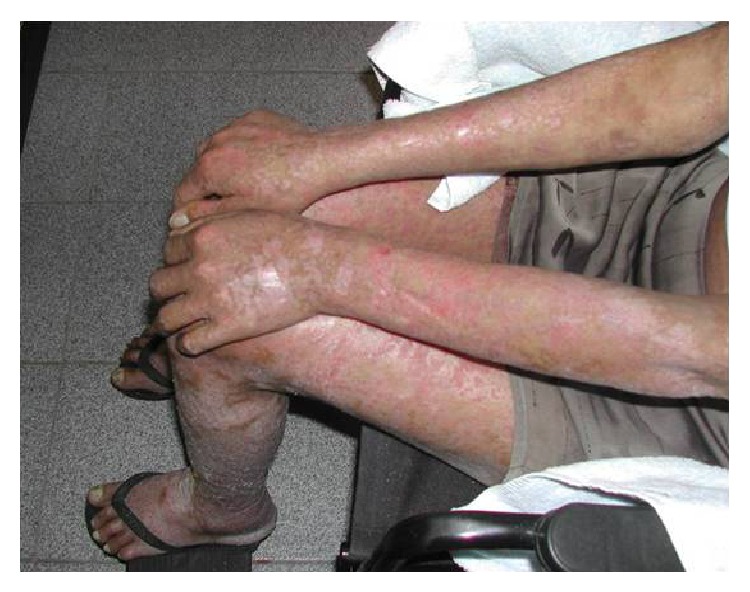
Desquamative and nonpruritic lesions in members.

**Figure 2:**
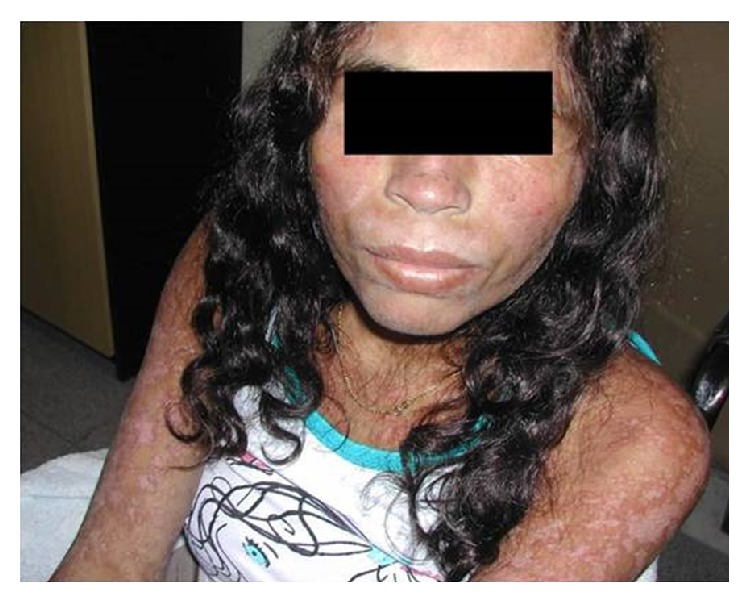
Desquamative and nonpruritic lesions in face.

**Figure 3:**
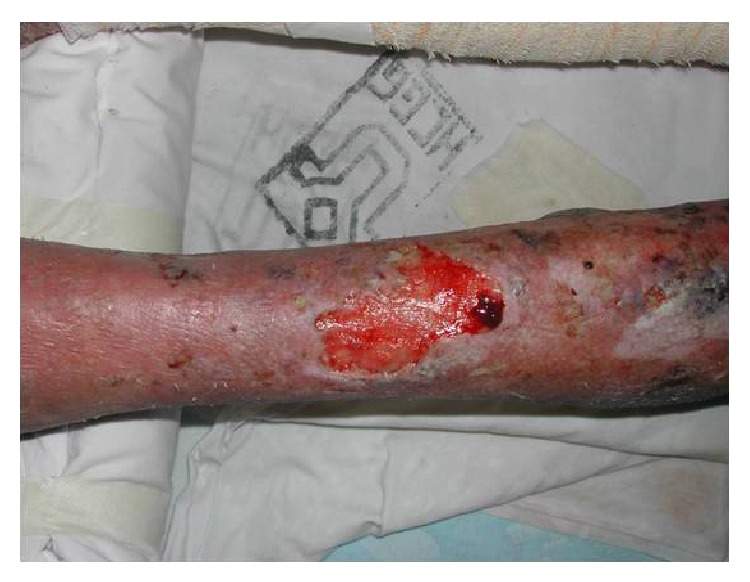
Ulcerated lesion with raised borders and sanguinary erythematous background in inferior member.

During hospitalization, there was widespread dissemination of lesions, especially in the lower limbs, and a tendency to confluence, evolving with scaly pruritic papules. After one month of evolution, the ulcerative lesions became painful, eliminating serosanguineous secretion (Figures 1, 2, and 3).

Additional tests showed a CD4^+^ T lymphocytes count of 51 cel/mm^3^. The blood count demonstrated anemia, with a hematocrit of 22.6% and hemoglobin 7.5 g/dL. Biopsy of lesions was requested and sent to culture and histopathological examination.

Due to complaints of nasal obstruction and pain, laryngoscopy was performed, showing no changes. A nasal endoscopy later revealed mucosal lesions with bilateral obstruction by infiltrating lesions. The lesions were biopsied and sent for histopathology and culture for bacteria, mycobacteria, and fungi research.

**Figure 4:**
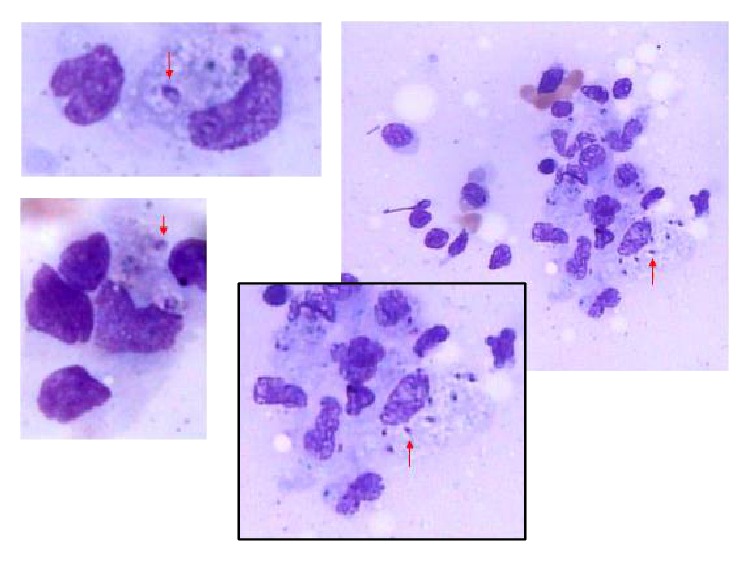
Numerous intracytoplasmic corpuscles of* Leishmania *spp. (red arrows) identified on histopathologic examination.

**Figure 5:**
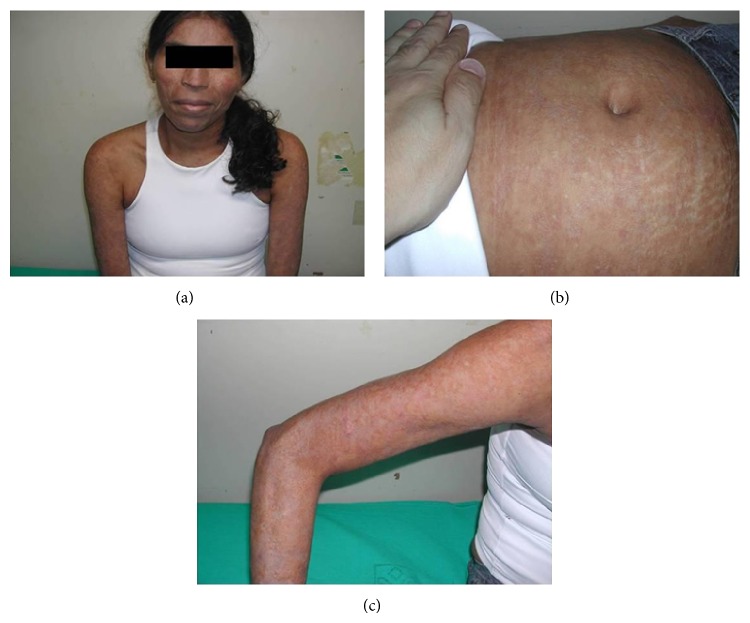
After 30 days of treatment, the lesions on the face (a), abs (b), and the upper limb (c) showed improvement.

Histopathology revealed numerous intracytoplasmic corpuscles (Figure 4), consistent with leishmaniasis. The culture identified* Leishmania (Viannia) braziliensis* as the causative agent of the infection. Specific treatment was started with antimonate N-methylglucamine (Glucantime) at a dose of 20 mg of pentavalent antimony (Sb)/kg/day, for which daily dose was 945 mg of Sb, dapsone 100 mg/day PO, and methadone 10 mg, 3x/day PO to relieve pain. The patient showed progressive improvement of the lesions (Figure 5), nasal obstruction, pain, and haematological parameters. She remained hospitalized for carrying out the specific medication treatment of leishmaniasis for 30 days. After this period, antiretroviral therapy was initiated with zidovudine + lamivudine (ATC) 300/150 mg PO 2x/day + efavirenz (EFV) 600 mg PO 1x/day and primary and secondary prophylaxis for* Pneumocystis *and for toxoplasmosis with orally sulfadiazine 4 g/day + pyrimethamine 25 mg/day VO + folinic acid 15 mg/day and the patient was discharged for outpatient monitoring.

## 3. Discussion

Leishmaniasis/HIV coinfection is considered an emerging problem in several countries, including Brazil, where, despite the growing number of cases, a problem of late diagnosis occurs worsening the prognosis [1]. Cases of leishmaniasis are more frequent in men, and 36% of the Brazilian cases have a previous diagnosis of HIV infection [8]. The published studies of leishmaniasis/HIV coinfection have specific interest in the VL, with more rare publications on the NVL. Series of Brazilian patients indicate that between 1982 and 2003 about 100 cases of leishmaniasis/HIV coinfection were published; 37% of cases corresponded to VL and 63% of cases corresponded to NVL. Of the NVL studies, 68% were MCL and 32% were LCL to DCL [13]. A subsequent review of 15 cases between 1998 and 2008 showed 13 cases of CL and 2 cases of MCL [16].

The leishmaniasis/HIV coinfection presents with peculiarities regarding their clinical manifestations. The cases of leishmaniasis associated with HIV infection may present with a wide spectrum from inadequate weight gain to extensive and destructive injuries [17].

Diffuse desquamative rash lesions, such as injuries presented by the patient, constitute a rare and atypical presentation of leishmaniasis/HIV coinfection, which could have been easily interpreted as adverse drug reactions. A relevant aspect is that leishmaniasis/HIV coinfection should be differentiated from forms that have numerous lesions and those without immunosuppression, such as those occurring in DCL and post-kala-azar dermal leishmaniasis (PKDL).

The DCL is characterized by more than ten lesions, has poor response to conventional treatment, presents as small papules, and mainly manifests as acneiform lesions. The spread occurs usually in 3 days to 8 weeks after the initial injury, arising between 10 and 800 secondary lesions [18, 19]. The PKDL is manifested by prominent skin lesions on the face, upper arms, and upper part of the trunk and can develop few months after treatment, with the possibility of also affecting the viscera [4]. PKDL is a dermatitis which tends to develop after treatment for VL in about 50% of cases in Sudan and 5 to 15% in India. In Sudan, the lesions usually develop during the treatment or within 6 months of VL treatment, whereas in Indian PKDL cases they appear after 2 to 3 years. PKDL has also been reported to develop even 10 years after VL treatment [20].

There is a possibility that the case described happened as a primoinfection that spread due to HIV-induced immunosuppression or by reactivation of an infection acquired in the past. The epidemiological risk of northeastern Brazil is higher than the region of Complexo do Alemão in Rio de Janeiro. However, in Rio de Janeiro NVL outbreaks occurred in Tijuca (1922), in Maciço da Pedra Branca (1973), and currently in Pau da Fome locality [21]. Reactivation occurs most commonly when the CD4^+^ T lymphocytes count falls below 200 cells/mm^3^. Up to 90% of cases have CD4^+^ T lymphocytes count with less than 200 cells/mm^3^ and in this case the patient had a count of 51 cells/mm^3^ [22, 23].

Despite the growing resistance of leishmaniasis to antimonial drugs, the patient had a good response to treatment. This case report draws attention to the clinical diversity presented by the leishmaniasis/HIV coinfection. Atypical presentations may delay diagnosis, especially in patients without prior diagnosis of HIV infection. Likewise, performing a diagnosis of leishmaniasis must evoke the screening of an HIV infection. The patient is performing outpatient treatment with full recovery of injuries and a special attention should be given to the possibility of recurrence.
